# Thyroid stimulating hormone suppression time on cardiac function of patients with differentiated thyroid carcinoma

**DOI:** 10.1186/s12935-018-0686-9

**Published:** 2018-11-19

**Authors:** Ruihua Wang, Liu Yang, Shui Jin, Xingmin Han, Baoping Liu

**Affiliations:** 1 Department of Nuclear Medicine, The First Affiliated Hospital, College of Medicine, Henan Medical Key Laboratory of Molecular Imaging, Zhengzhou University, 1 Jianshe East Road, Zhengzhou, 450052 Henan China; 2 Department of Nuclear Medicine, Zhejiang Cancer Hospital, Hangzhou, 310011 Zhejiang China

**Keywords:** Thyroid stimulating hormone, Differentiated thyroid carcinoma, Cardiac function, Suppression time, Myocardial perfusion

## Abstract

**Background:**

This study was to investigate the influence of thyroid stimulating hormone (TSH) suppression time on the cardiac function of differentiated thyroid carcinoma (DTC) patients.

**Methods:**

105 DTC patients were divided into strict TSH suppression group (model group, TSH ≤ 0.1 mU/L) and general TSH suppression group (control group, TSH > 0.1 mU/L). According to the suppression time, these two groups were respectively divided into three groups: group within half a year, group between half a year and a year and group more than a year. Gated myocardial perfusion imaging was applied to observe differences of left ventricle (LV) myocardial perfusion, LV diastolic and systolic function and LV systolic synchrony in every group.

**Results:**

The left ventricular diastolic function, systolic synchrony and myocardial perfusion level of model group decreased with prolonged suppression time. The values of left ventricular EF, PFR and BPM in patients less than half a year were higher than those in 6 months to 1 year for control group.

**Conclusion:**

Thyroid stimulating hormone suppression can influence the cardiac function of patients and with the prolongation of suppression time, regardless of the level of TSH suppression, the possibility of cardiac function depression in patients will increase. TSH may lower the risk of cardiovascular disease in high-risk patients than those in TSH patients with moderate or low risk. The drugs improving cardiac function should be used cooperatively in different suppression period to decrease the occurrence rate of cardiac adverse reactions.

## Background

Differentiated thyroid carcinoma (DTC) is a common malignant tumor affecting endocrine system and accounts for 90% of thyroid carcinomas [1, 2]. Thyroid stimulating hormone (TSH) suppression is a key step for DTC treatment and has been applied wildly in clinical practice. However, long-term TSH suppression may lead to an exogenous subclinical hyperthyroidism and increase thyroxine (T4) level and T4/triiodothyronine (T3) level [3], which may induce many adverse reactions and negative effects on cardiac function [4–6]. At present, the specific TSH suppression level to the best therapeutic effect is controversial. A research in Japan showed no difference in disease free survival between patients with TSH suppression < 0.1 mU/L and patients with TSH suppression > 0.1 mU/L. However, other research showed TSH suppression < 0.1 mU/L could significantly improve the prognostic recovery of patients with high-risk thyroid cancer [7, 8]. In this investigation, gated myocardial perfusion imaging (GMPI) [9] was applied to observe the influence of different TSH suppression levels and TSH suppression time on cardiac function from the perspective of left ventricle (LV) myocardial perfusion, LV diastolic and systolic function and LV systolic synchrony so as to provide a theoretical basis for the use of different drugs which can improve cardiac function in different treatment period.

## Methods

### Patients

105 DTC patients in our hospital from October 2014 to January 2016 were collected. This study was approved by the ethic committee of First Affiliated Hospital of Zhengzhou University. All patients had no history of heart disease or related diseases; all patients had not received any medication that might affect heart function. The data were analyzed anonymously. After total thyroidectomy, ^131^I treatment and TSH suppression (l-thyroxine) were performed. Free triiodothyronine (FT3), free thyroxine (FT4) and TSH level in patients’ serum were tested every 2 or 3 months (Table 1).

**Table 1:** Thyroid function test results before and after TSH treatment

	Before TSH treatment	After TSH treatment	P
TSH (mU/L)	2.01 ± 0.08	4.66 ± 0.93	0.003
T3 (nmol/L)	1.67 ± 0.85	2.23 ± 0.44	0.051
FT3 (pmol/L)	3.16 ± 0.94	4.40 ± 0.84	0.059
T4 (nmol/L)	67.96 ± 0.63	71.16 ± 0.54	0.050
FT4 (pmol/L)	11.06 ± 0.41	12.95 ± 0.33	0.331

*TSH* thyroid stimulating hormone, *T3* triiodothyronine, *FT3* free triiodothyronine, *T4* thyroxine, *FT4* free thyroxine

According to the standard proposed by the American Thyroid Association [10], patients with high-risk thyroid cancer were recommended a TSH suppression ≤ 0.1 mU/L, and moderate- and low- risk patients were recommended a TSH suppression > 0.1 mU/L. According to TSH level in serum, all patients were divided into strict TSH suppression group (model group, TSH ≤ 0.1 mU/L, 64 patients) and general TSH suppression group (control group, TSH > 0.1 mU/L, 41 patients). In model group, the average age was 46.56 ± 9.16, average treatment time was 0.99 ± 0.71 (year). In control group, the average age was 45.92 ± 10.89, average treatment time was 1.05 ± 1.09 (year). There was no statistical difference in both two groups (*P *> 0.05).

According to TSH suppression time (within half a year, between half a year and a year and more than a year), these two groups above were respectively divided into three groups and the influence of suppression time on cardiac function was studied. The model group was divided into group A (within half a year, 5 patients), group B (between half a year and a year, 21 patients) and group C (more than a year, 28 patients). The control group was divided into group D (within half a year, 17 patients), group E (between half a year and a year, 8 patients) and group F (more than a year, 16 patients).

### Instruments and examination indexes

#### TSH determinator

Electrochemistry luminescence immunity analyzer (Cobase 411, Roche, Germany). Electrochemical luminescence detection kit was also bought from Roche Company.

#### GMPI machine

It was used for the assessment of patients’ cardiac function. SPECT (Symbia-T16, Siemens, Germany) was used for scanning. 99mTc-MIBI was used, radiochemical purity > 95%. Imaging agent was bought from HTA CO. LTD.

### Main determined indexes of cardiac function

#### Myocardial perfusion indexes

Summed rest score (SRS) and myocardial perfusion target chart.

#### Cardiac diastolic and systolic functions indexes

Beats per minute (BMP), LV end-diastolic volume (EDV), LV end-systolic volume (ESV), left ventricular ejection fraction (LVEF), peak ejection rate (PER), peak filling rate (PFR), mean filling rate over the first third of the end-systolic to end diastolic phase (MFR/3), time to peak filling from end-systole (TTPF) and SRS.

#### Cardiac phase analysis indexes

Bandwidth, mean angle of LV myocardium systolic, angle standard deviation (STD) of LV myocardium systolic and entropy of LV myocardium systolic.

### Statistical analysis

All statistical analyses were performed using SPSS version 19.0 software. Independent-samples T test was performed for the comparison of cardiac function data in both model group and control group. ANOVA was used for multiple group comparision. One-way analysis of variance was performed for the comparison in every two groups with different suppression time. There was a significant difference at *P *< 0.05.

## Results

### Cardiac function data comparison decreased with the increase of TSH suppression time in model group (TSH strict suppression group)

Cardiac function data comparison in model group was showed in Table 2. Compared with group B, ejection fraction (EF) value in group A was lower, the difference was statistically significant (*P *< 0.05), there was no notable difference on other cardiac function indexes. Compared with group C, in group A, LVPER and LVPFR were higher (*P *< 0.05), TTPF was shorter (*P *< 0.05), bandwidth and entropy of LV myocardium systolic were smaller (*P *< 0.05). Compared with group C, in group B, EF value, LVPER, LVPFR and MFR/3 were higher, bandwidth and entropy of LV myocardium systolic were smaller (*P *< 0.05). The perfusion reduction rate increased with the extension of suppression time. All of above showed that in model group, suppression time had influence on cardiac function. The ventricular synchrony was significantly inhibited when suppression time was more than 1 year.

**Table 2:** Cardiac function data comparison in model group with different suppression time

	Group A: within half a year (15 patients)	Group B: between half a year and a year (21 patients)	Group C: more than a year (28 patients)	P_AB_	P_AC_	P_BC_
EF (%)	73.01 ± 5.18	78.66 ± 5.03	71.39 ± 8.72	0.049	0.274	0.002
PER (EDV/s)	3.97 ± 0.85	4.23 ± 0.44	3.23 ± 0.60	0.438	0.001	0.000
PFR (EDV/s)	3.66 ± 1.04	3.40 ± 0.81	2.38 ± 0.82	0.589	0.000	0.001
MFR/3 (EDV/s)	1.66 ± 0.63	1.66 ± 0.44	1.36 ± 10.29	0.999	0.090	0.050
TTPF (ms)	149.06 ± 46.41	160.95 ± 23.33	172.32 ± 13.75	0.412	0.028	0.331
BMP	80.80 ± 12.09	77.52 ± 7.37	75.44 ± 12.50	0.652	0.286	0.789
Bandwidth	24.11 ± 11.92	25.95 ± 12.59	35.11 ± 18.37	0.869	0.006	0.012
Mean angle (°)	147.52 ± 9.71	144.13 ± 10.97	143.15 ± 17.27	0.751	0.590	0.968
Angle STD (°)	8.64 ± 6.75	8.00 ± 5.59	11.85 ± 10.47	0.972	0.454	0.252
Entropy (%)	27.26 ± 11.86	28.28 ± 8.88	36.92 ± 10.30	0.953	0.013	0.013
SRS	1.20 ± 1.42	0.95 ± 1.32	2.88 ± 2.00	0.898	0.007	0.001
Reduced perfusion ratio (%)	16.67	33.33	67.86	–	–	–

*EF* ejection fraction, *PER* peak ejection rate, *EDV* end-diastolic volume, *PFR* peak filling rate, *MFR/3* mean filling rate over the first third of the end-systolic to end diastolic phase, *TTPF* time to peak filling from end-systole, *BMP* beats per minute, *STD* standard deviation, *SRS* summed rest score

### Cardiac function data comparison decreased with the increase of TSH suppression time in control group (TSH general suppression group)

Cardiac function data comparison in control group was showed in Table 3. There was no notable difference on the data of LV diastolic and systolic function, myocardial perfusion level and myocardial systolic synchrony in group D and group E. Compared with group F, LVEF value, LVPFR, BMP and SRS were higher in group D (*P *< 0.05). There was no notable difference on the data of LV diastolic and systolic function, myocardial perfusion level and myocardial systolic synchrony in group E and group F. All of above showed that in control group, suppression time had influence on cardiac function. The cardiac function was significantly inhibited when suppression time was more than 1 year, increasing the risk of cardiovascular disease.

**Table 3:** Cardiac function data comparison in control group with different suppression time

	Group D: within half a year (17 patients)	Group E: between half a year and a year (8 patients)	Group F: more than a year (16 patients)	P_DE_	P_DF_	P_EF_
EF (%)	75.05 ± 9.60	72.50 ± 5.63	57.61 ± 7.75	0.798	0.030	0.037
PER (EDV/s)	4.01 ± 0.86	3.81 ± 0.36	2.66 ± 0.82	0.857	0.401	0.035
PFR (EDV/s)	3.71 ± 0.90	3.22 ± 0.52	2.00 ± 0.73	0.395	0.028	0.004
MFR/3 (EDV/s)	1.36 ± 0.41	1.68 ± 0.20	0.55 ± 0.31	0.138	0.246	0.006
TTPF (ms)	156.94 ± 28.01	153.33 ± 33.59	117.77 ± 13.30	0.945	0.994	0.016
BMP	88.17 ± 7.98	77.15 ± 7.23	52.97 ± 13.80	0.098	0.001	0.010
Bandwidth	22.82 ± 17.29	39.94 ± 29.22	22.89 ± 8.80	0.087	0.423	0.019
Mean angle (°)	156.06 ± 14.21	142.33 ± 14.27	101.15 ± 18.30	0.187	0.060	0.004
Angle STD (°)	5.40 ± 6.32	10.63 ± 10.61	7.02 ± 6.77	0.291	0.786	0.544
Entropy (%)	22.88 ± 10.51	33.33 ± 14.79	21.94 ± 12.95	0.185	0.448	0.013
SRS	0.82 ± 0.48	0.67 ± 1.63	1.67 ± 1.88	0.973	0.195	0.307
Reduced perfusion ratio (%)	11.76	16.67	61.11	–	–	–

*EF* ejection fraction, *PER* peak ejection rate, *EDV* end-diastolic volume, *PFR* peak filling rate, *MFR/3* mean filling rate over the first third of the end-systolic to end diastolic phase, *TTPF* time to peak filling from end-systole, *BMP* beats per minute, *STD* standard deviation, *SRS* summed rest score

### Strict suppression of TSH can inhibit cardiac function in patients within suppression time more than a year

Data analysis was performed in group A and group D, group B and group E, group C and group F, respectively, so as to further study the different influence on cardiac function with the same suppression time in both model group and control group. As for myocardial perfusion level, the occurrence rate of myocardial perfusion reduction in model group was higher than that in control group in spite of suppression time. SRS difference in the suppression time within half a year and more than a year showed statistical significance (*P *< 0.05). As for other cardiac function, there was no notable difference on systolic and diastolic function and phase analysis in both model group and control group with the same suppression time (within half a year). For the suppression time between half a year and a year, LVEF value and PER were higher in model group than those in control group (*P *< 0.05) (Table 4). When the suppression time was more than a year, LVPER, LVPFR and MFR/3 decreased in both model group and control group, TTPF was also delayed. In model group, the bandwidth and entropy of LV myocardium systolic were higher than that in control group (*P *< 0.05) (Table 5). Cardiac function data comparison in model group and control group before TSH suppression treatment has no difference (Table 6).

**Table 4:** Cardiac function data comparison in group B and group E

	Group B (21 patients)	Group E (6 patients)	P
EF (%)	78.66 ± 5.03	72.50 ± 5.63	0.016
PER (EDV/s)	4.23 ± 0.44	3.81 ± 0.36	0.043
Reduced perfusion ratio (%)	33.33	16.67	–

*EF* ejection fraction, *PER* peak ejection rate, *EDV* end-diastolic volume

**Table 5:** Cardiac function data comparison in group C and group F

	Group C (28 patients)	Group F (18 patients)	P
PER (EDV/s)	3.23 ± 0.60	3.66 ± 0.82	0.046
PFR (EDV/s)	2.38 ± 0.82	3.00 ± 0.73	0.041
MFR/3 (EDV/s)	1.36 ± 10.29	1.55 ± 0.31	0.040
TTPF (ms)	172.32 ± 13.75	157.77 ± 13.30	0.001
Bandwidth	35.11 ± 18.37	29.89 ± 8.80	0.049
Entropy (%)	36.92 ± 10.30	27.94 ± 12.95	0.012
SRS	2.88 ± 2.00	1.67 ± 1.88	0.046
Reduced perfusion ratio (%)	67.86	61.11	–

*PER* peak ejection rate, *EDV* end-diastolic volume, *PFR* peak filling rate, *MFR/3* mean filling rate over the first third of the end-systolic to end diastolic phase, *TTPF* time to peak filling from end-systole, *SRS* summed rest score

**Table 6:** Cardiac function data comparison in model group and control group before TSH suppression treatment

	Model group (n = 64)	Control group (n = 41)	*P*
EF (%)	83.01 ± 5.08	82.66 ± 5.53	0.073
PER (EDV/s)	4.67 ± 0.85	4.23 ± 0.44	0.051
PFR (EDV/s)	5.16 ± 0.94	5.40 ± 0.84	0.059
MFR/3 (EDV/s)	1.96 ± 0.63	2.16 ± 0.54	0.050
TTPF (ms)	179.06 ± 36.41	170.95 ± 23.33	0.331
BMP	90.80 ± 11.29	87.62 ± 6.37	0.789
Bandwidth	27.11 ± 11.42	28.95 ± 10.49	0.052
Mean angle (°)	201.52 ± 9.31	203.13 ± 8.97	0.968
Angle STD (°)	9.34 ± 6.01	9.00 ± 4.19	0.252
Entropy (%)	30.26 ± 10.26	29.28 ± 8.91	0.013
SRS	1.10 ± 0.42	0.99 ± 0.32	0.001
Reduced perfusion ratio (%)	26.67	29.03	–

*EF* ejection fraction, *PER* peak ejection rate, *EDV* end-diastolic volume, *PFR* peak filling rate, *MFR/3* mean filling rate over the first third of the end-systolic to end diastolic phase, *TTPF* time to peak filling from end-systole, *BMP* beats per minute, *STD* standard deviation, *SRS* summed rest score

## Discussion

Cardiac function data comparison was performed in group A, group B and group C. The result shows that LVEF value and systolic function increased at the early stage and decreased at later stage, while LV diastolic function, myocardial systolic synchrony and myocardial perfusion level decreased with the extension of suppression time. Then cardiac function data comparison was performed in group D, group E and group F. The result shows that when suppression time was within half a year, LVEF, LVPFR and BMP were higher than that with the suppression time between half a year and a year, which indicates that except for TSH suppression, there is still another factor which has influence on cardiac function. However, comparing model group with control group, we can find that TSH suppression is still the main influence factor for DTC patients, especially for patients with long-term suppression. However, strict TSH suppression can inhibit patients’ cardiac function, which indicates that high-risk patients with TSH suppression have a higher occurrence rate of cardiovascular disease than moderate- and low- risk patients with TSH suppression.

At the early stage of suppression (within half a year), there was no notable difference on myocardial perfusion level in both control group and model group. When the suppression time was more than a year, notable difference on myocardial perfusion level appeared, and LV myocardial perfusion level decreased in model group. Among patients with suppression time between half a year and a year, LVEF value and PER were higher in model group than that in control group. The possible reason may be that when TSH suppression was performed, high physiological doses of exogenous thyroid hormone were used to improve cardiac load through improving the activity of Ca-ATPase in sarcoplasmic reticulum. At the early stage, it performed as compensatory increase of LV systolic function and the increase of cardiac output function [11]. When the suppression time was more than a year, cardiac reserve capacity was declined. Thus LVPER in model group was lower than that in control group.

The influence of suppression treatment on LV diastolic function is a long-term course. At the early stage of suppression (within half a year), there was no evidence showed any difference on LV diastolic function in both model group and control group. However, when the suppression time was more than a year, PFR, MFR/3 and TTPF all decreased in model group. The same result showed in the comparison of LV phase analysis, when the suppression time was more than a year, systolic synchronicity data in model group (bandwidth and entropy of LV myocardium systolic) were all higher than that in control group. All of these show that long-term strictly TSH suppression (more than a year) has greater influence on LV diastolic function and systolic synchronicity than that with general TSH suppression.

## Conclusions

This investigation shows that with different suppression time, the influence of TSH suppression on cardiac function is different. The concrete difference needs further study. At present, the commonly used l-thyroxine is a main method of TSH suppression for patients with DTC. The positive effects of l-thyroxine are heart rate acceleration, shortening of myocardial refractory period, conduction of electrocardiogram acceleration, and increasing cardiac synchrony. In clinical treatment, drugs which can improve cardiac function should be used in different suppression period, so as to improve the cardiac hemodynamics, stabilize ventricular rate, decrease the occurrence of thromboembolism caused by atrial fibrillation, reduce the rate and seriousness of paroxysmal atrial fibrillation and improve the cardiac systolic and diastolic function [12–14]. For example, the combination of β-blocker and TSH suppression has effect for the atrial arrhythmia and diastolic dysfunction caused by TSH suppression [15] and reduces the death rate caused by adverse cardiovascular reactions [16–18]. The combination of atenolol [19], digoxin, angiotensin converting enzyme inhibitor and other cardiovascular drugs can effectively reduce the adverse cardiovascular reactions during the suppression process [20]. Some studies have showed that TSH levels were associated in a positive and linear manner with the TC, non-HDL-C and TG levels in euthyroid non-diabetics with newly diagnosed asymptomatic CHD. TSH in the upper limits of normal range might exert adverse effects on the lipid profile and might represent a risk factor for hypercholesterolaemia and hypertriglyceridaemia in the context of CHD. Maintaining TSH in a relative low normal range might be beneficial for lipid profile in euthyroid non-diabetics with newly diagnosed asymptomatic CHD [21]. At present, this study only applies to specific groups of people, and has not yet included dietary factors in the study, so the conclusion can not be extended to other groups; our next study is to expand the sample size of multi-center study.

## Abbreviations

TSH: thyroid stimulating hormone
DTC: differentiated thyroid carcinoma
LV: left ventricle
T4: thyroxine
T3: triiodothyronine
GMPI: gated myocardial perfusion imaging
FT3: free triiodothyronine
FT4: free thyroxine
SRS: summed rest score
BMP: beats per minute
EDV: end-diastolic volume
ESV: end-systolic volume
LVEF: left ventricular ejection fraction
PER: peak ejection rate
PFR: peak filling rate
MFR: mean filling rate
TTPF: time to peak filling from end-systole
STD: standard deviation
EF: ejection fraction
TC: total cholesterol
CHD: coronary heart disease
TG: triglycerides
Non-HDL-C: non-high density lipoprotein cholesterol
